# YiXin-Shu, a ShengMai-San-based traditional Chinese medicine formula, attenuates myocardial ischemia/reperfusion injury by suppressing mitochondrial mediated apoptosis and upregulating liver-X-receptor α

**DOI:** 10.1038/srep23025

**Published:** 2016-03-11

**Authors:** Yichao Zhao, Longwei Xu, Zhiqing Qiao, Lingchen Gao, Song Ding, Xiaoying Ying, Yuanyuan Su, Nan Lin, Ben He, Jun Pu

**Affiliations:** 1 Department of Cardiology, Ren Ji Hospital, School of Medicine, Shanghai Jiao Tong University. 160 PuJian Road, Shanghai 200127, China

## Abstract

Positive evidence from clinical trials has fueled growing acceptance of traditional Chinese medicine (TCM) for the treatment of cardiac diseases; however, little is known about the underlying mechanisms. Here, we investigated the nature and underlying mechanisms of the effects of YiXin-Shu (YXS), an antioxidant-enriched TCM formula, on myocardial ischemia/reperfusion (MI/R) injury. YXS pretreatment significantly reduced infarct size and improved viable myocardium metabolism and cardiac function in hypercholesterolemic mice. Mechanistically, YXS attenuated myocardial apoptosis by inhibiting the mitochondrial mediated apoptosis pathway (as reflected by inhibition of mitochondrial swelling, cytochrome *c* release and caspase-9 activity, and normalization of Bcl-2 and Bax levels) without altering the death receptor and endoplasmic reticulum-stress death pathways. Moreover, YXS reduced oxidative/nitrative stress (as reflected by decreased superoxide and nitrotyrosine content and normalized pro- and anti-oxidant enzyme levels). Interestingly, YXS upregulated endogenous nuclear receptors including LXRα, PPARα, PPARβ and ERα, and *in-vivo* knockdown of cardiac-specific LXRα significantly blunted the cardio-protective effects of YXS. Collectively, these data show that YXS is effective in mitigating MI/R injury by suppressing mitochondrial mediated apoptosis and oxidative stress and by upregulating LXRα, thereby providing a rationale for future clinical trials and clinical applications.

Ischemic heart disease remains one of the leading causes of death worldwide^1^,^2^,^3^. Myocardial ischemia can lead to myocardial damage and heart dysfunction, which can be restored through reperfusion. However, myocardial reperfusion might trigger additional injury, known as myocardial ischemia reperfusion (MI/R) injury^4^,^5^,^6^. Acute MI/R injury is associated with excessive accumulation of reactive oxygen species, augmented myocardial apoptosis, and increased infarct size which significantly contribute to long-term mortality and chronic heart failure^7^. Therefore, strategies limiting MI/R injury extent, albeit currently limited, are of great clinical and health value.

In recent years, positive evidence from clinical trials has favored acceptance of TCM^8^ for the treatment of cardiac diseases^9^,^10^,^11^. Based on the notion that mixed herbal ingredients hit multiple targets with synergistic effects and less toxicity than one ingredient^12^, TCM medications generally incorporate herbs with so called sovereign (major active components), minister (synergistic activities), and assistant (detoxification) roles^10^. ShengMai-San (SMS), one of the most ancient TCM formulas and consisting of *Radix Ginseng* (sovereign), *Radix Ophiopogonis* (minister), and *Fructus Schisandrae Chinensis* (assistant), protects tissues from oxidative damage in heart disease, cerebral injury and carbon tetrachloride induced hepatic damage^13^. YiXin-Shu (YXS), a SMS-derived TCM formula that was specifically developed for the treatment of ischemic heart disease^14^, contains four more herbs, namely *Salvia Miltiorrhiza* (sovereign), *Astragalus Membranaceus* (minister), *Ligusticum Wallichii* (minister), and *Fructus Crataegi* (assistant) (Supplementary Table S1) with potent effects on intracellular calcium handling^15^, mitochondrial oxidative phosphorylation^16^ and neovascularization^17^ that are complementary to the pharmacological effects of SMS components^18^,^19^,^20^ and therefore thought to augment cardio-protective activity. Although YXS is widely used in Asia for the treatment of coronary artery disease, data are lacking on the nature and underlying mechanisms of its beneficial effects, especially in acute MI/R injury, which is the subject of the present study in hypercholesterolemic mice.

## Results

### YXS reduces MI/R-induced infarct size and cardiac dysfunction

**Figure 1:**
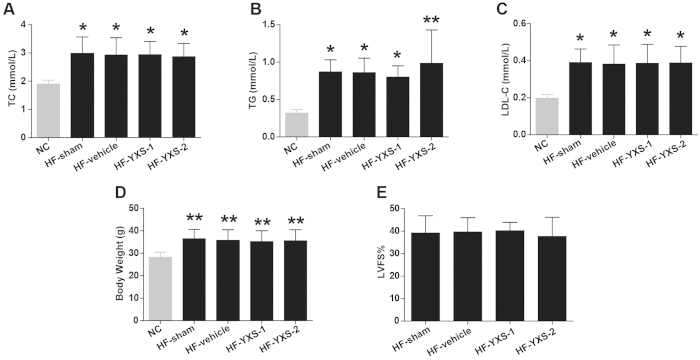
Baseline lipid profile and cardiac function among treatment groups. (**A–C**) Plasma levels of TC (**A**) TG (**B**) and LDL-C (**C**) were determined in NC, HF-sham, HF-vehicle, HF-YXS-1, HF-YXS-2 groups by an auto-biochemical analysis system (n = 5 animals per group). (**D**) Body weight was recorded in indicated groups (n = 10 animals per group). (**E**) Baseline LVFS was measured by echocardiography before induction of MI/R injury in all groups (n = 6 animals per group). *p < 0.05 or **p < 0.01 versus NC. Abbreviations: TC, total cholesterol; TG, triglyceride; LDL-C, low-density lipoprotein-cholesterol; NC, normal diet fed group; HF-sham, high-cholesterol diet fed group; HF-vehicle, high-cholesterol diet fed group followed by saline treatment; HF-YXS-1, high-cholesterol diet fed group followed by YXS (60 mg·kg^−1^·d^−1^) treatment; HF-YXS-2, high-cholesterol diet fed group followed by YXS (120 mg·kg^−1^·d^−1^) treatment.

**Figure 2:**
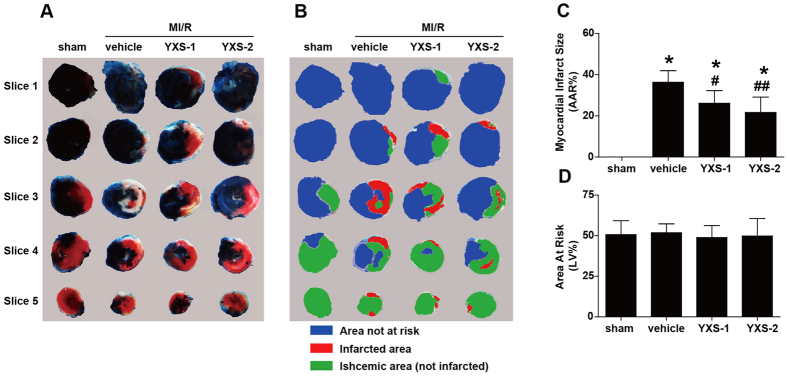
YXS pretreatment reduces myocardial infarct size. (**A**) Representative photographs of Evans blue/TTC double stained murine heart slices obtained 24 h after MI/R injury. (**B**) Distribution of ischemic area, infarcted area and area not at risk in each slice. (**C,D**) Graphic representation of the LV infarct size (**C**) and AAR (**D**) (n = 6–8 animals per group). *p < 0.01 versus sham; ^**#**^*p* < 0.05 or ^**##**^*p* < 0.01 versus vehicle. Abbreviation: AAR, area at risk.

**Figure 3:**
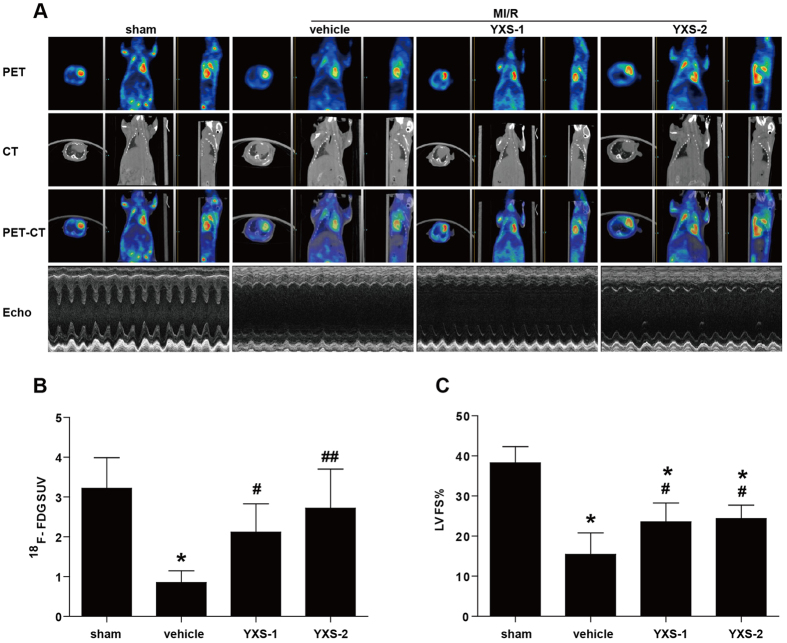
YXS pretreatment improves viable myocardium metabolism and cardiac function. (**A**) Representative images of Micro-PET, Micro-CT, Micro-PET/CT overlap, and echocardiography. Viable myocardium metabolism was assessed via ^18^F-FDG uptake by Micro-PET/CT, and cardiac performance was determined by echocardiography 24 h after reperfusion. (**B**) Myocardial SUV of ^18^F-FDG determined by ^18^F-FDG uptake utilizing Micro-PET/CT (n = 5 animals per group). (C) LVFS values were measured by echocardiography (n = 5–6 animals per group). *p < 0.01 versus sham; ^**#**^*p* < 0.05 or ^**##**^*p* < 0.01 versus vehicle. Abbreviations: SUV, standardized uptake value; LVFS, left ventricular fractional shortening.

To investigate the effects of YXS on MI/R-induced injury in hypercholesterolemic mice, mice were fed with high-cholesterol diets for 8 weeks, and subsequently randomly assigned to the following groups: sham, vehicle (saline), YXS-1 (60 mg·kg^−1^·d^−1^, equivalent to clinical dosage), or YXS-2 (120 mg·kg^−1^·d^−1^) for 1 week. High-cholesterol diets led to significantly higher levels of plasma TC, TG, LDL-C, and body weight, while YXS pretreatment did not affect plasma lipid profile or body weight (Fig. 1A–D) or cardiac performance at baseline (Fig. 1E). Following MI/R, YXS significantly reduced infarct size [36.3 ± 5.6% in vehicle group, vs. 26.1 ± 6.1% in YXS-1 group (P < 0.05), and 21.7 ± 7.4% in YXS-2 group (P < 0.01), Fig. 2A–C], while AARs were similar among treatment groups (Fig. 2D). ^18^F-FDG micro-PET/CT scanning and echocardiography were performed to determine the effects of YXS on viable myocardium metabolism and cardiac performance. Compared to sham, MI/R significantly reduced mean myocardial SUV of ^18^F-FDG, and impaired contractile function (Fig. 3A). By contrast, YXS treatment significantly increased ^18^F-FDG uptake [0.85 ± 0.13 in the vehicle group, vs. 2.12 ± 0.71 in the YXS-1 group (P < 0.05) and 2.72 ± 0.98 in the YXS-2 group (P < 0.01), Fig. 3B], and attenuated MI/R-induced impairment of LVFS [15.4 ± 5.3% in vehicle group, vs. 23.6 ± 4.7% in YXS-1 group (P < 0.05) and 24.4 ± 3.4% in YXS-2 group (P < 0.05), Fig. 3D] compared with vehicle treatment. Collectively, these data suggest that YXS pretreatment reduces infarct size and improves myocyte viability and cardiac performance in a murine model of MI/R injury.

### YXS suppresses mitochondrial-mediated apoptosis

**Figure 4:**
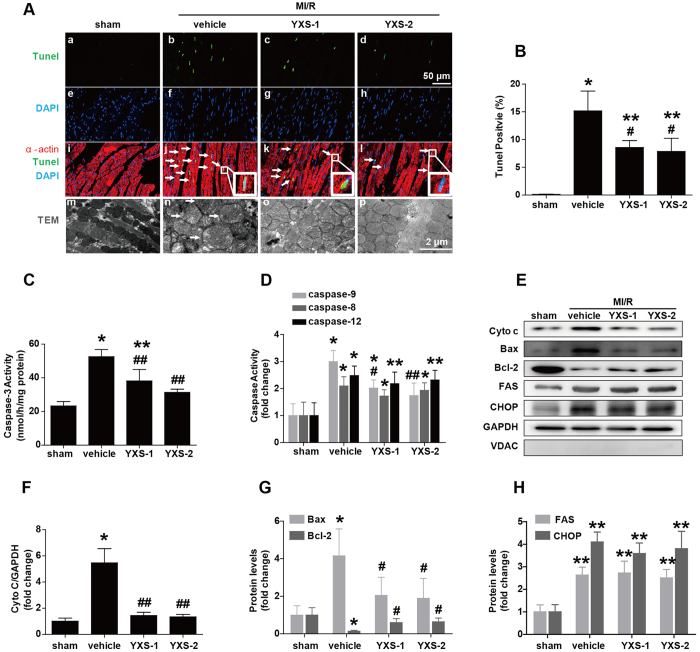
YXS pretreatment suppresses the mitochondrial-mediated apoptosis pathway. (**A**) Representative photomicrographs of TUNEL staining (a–l) and transmission electron microscopy images (m–p). MI/R led to significant apoptosis (arrows) and mitochondrial swelling (arrows). Total nuclei were labeled with DAPI (blue), α-actin was labeled in red, and apoptotic nuclei were detected by TUNEL staining (green). (**B**) Quantitative analysis of apoptotic cells in indicated groups (n = 6 animals per group, n = 4 in sham). (**C,D**) Activities of caspase-3 **(C**) and caspase-8/-9/-12 (**D**) were measured through the specific cleavage of respective substrates in each group (n = 5 animals per group, n = 4 in sham). (**E**) Protein expression levels of cytosolic Cyto c, Bax, Bcl-2, FAS and CHOP in ischemic/reperfused myocardial tissue were determined by Western blot analysis. The absence of VDAC, a mitochondrial marker, in the cytosolic fraction verified that expression of cytosolic Cyto-C represented specific mitochondrial Cyto-C release. (**F–H**) Background-subtracted density of the bands for Cyto c, Bax, Bcl-2, FAS and CHOP was normalized against GAPDH and expressed as fold change relative to sham-operated controls (n = 5 animals per group). *p < 0.05 or **p < 0.01versus sham; ^#^*p* < 0.05 or ^##^*p* < 0.01 versus vehicle. Cropped blots were used here and the full-length gels were included in the supplementary information.

Myocardial apoptosis is an important contributor to infarct size expansion and cardiac dysfunction after ischemic reperfusion injury^21^,^22^. Therefore, we performed TUNEL staining to investigate whether YXS prevented MI/R-induced myocardial apoptosis. Following MI/R, vehicle-treated mice exhibited obvious cell apoptosis in ischemic myocardium compared to sham treated mice (Fig. 4A). By contrast, a significantly lower proportion of apoptotic cells was observed in heart slices from YXS-treated mice (Fig. 4B). Furthermore, as shown in Fig. 4C, YXS dramatically decreased the activity of caspase-3 (a key executor of apoptosis). These data suggest a potent anti-apoptotic effect of YXS in MI/R injury.

Three apoptosis pathways including the death receptor pathway, the mitochondrial-mediated apoptosis pathway and the endoplasmic reticulum (ER)-stress related apoptosis pathway, are involved in mediating the apoptotic cascade in ischemic heart injury^22^. We further tested the effects of YXS on activities of caspase-8 (as index for the death receptor pathway), caspase-9 (as index for the mitochondrial-mediated apoptosis pathway) and caspase-12 (as index for the ER-stress pathway). Following MI/R, caspase-8, -9, and -12 were all significantly activated (Fig. 4D), while YXS administration significantly inhibited caspase-9 activity, an index of mitochondrial-mediated apoptosis pathway, without altering caspase-8 and caspase-12 activities (Fig. 4D). Consistently, YXS inhibited mitochondrial swelling (Fig. 4A, bottom panel) and reduced mitochondrial cytochrome *c* (Cyto-c) release to cytoplasm (Fig. 4E,F), which is a key step in initiating mitochondrial-mediated apoptosis^22^. In addition, YXS treatment normalized the expression of Bax and Bcl-2 (Fig. 4G), two important apoptosis regulatory factors implicated in mitochondrial-mediated apoptosis^22^. By contrast, YXS treatment did not significantly alter the expression of CHOP (a mediator of the ER-stress apoptosis pathway) and FAS (a mediator of the death receptor pathway) (Fig. 4H). Taken together, these data suggest that YXS maintains cardiac survival by mainly suppressing the mitochondrial-mediated apoptosis pathway.

### YXS attenuates oxidative stress in ischemic/reperfused myocardium

**Figure 5:**
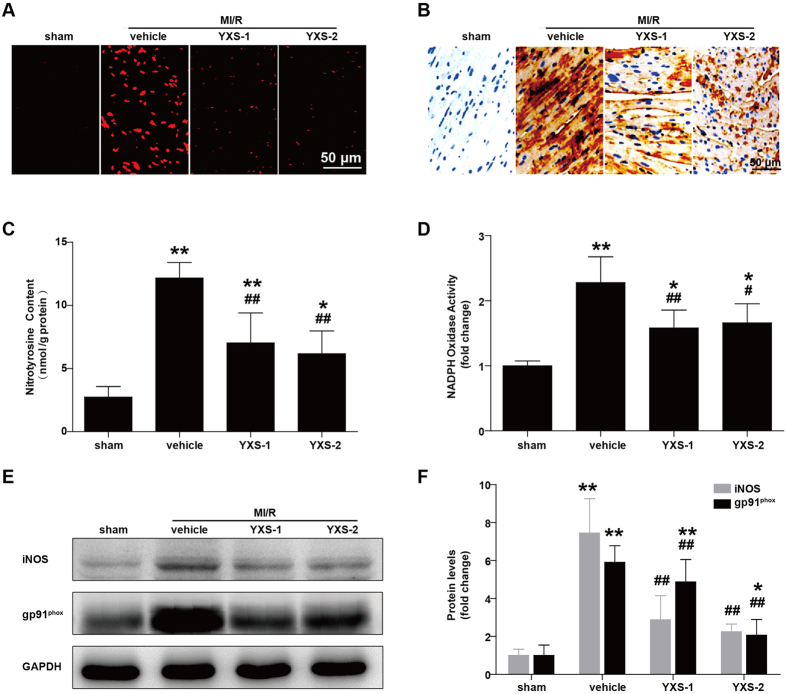
YXS pretreatment reduces MI/R-induced oxidative stress. (**A**) ROS steady-state levels in ischemic myocardium were measured by *in-situ* dihydroethidium staining, and images were obtained with confocal microscopy. (**B**,**C**) Nitrotyrosine production in ischemic/reperfused myocardium was measured by immunohistochemistry (**B**) and ELISA (**C**, n = 5 animals per group). (**D**) NADPH oxidase activity was measured by lucigenin-enhanced chemiluminescence, and expressed as fold change relative to sham-operated controls (n = 5 animals per group). (**E,F**) Protein levels of iNOS and gp91^phox^ were detected by Western blot (**E**) and subjected to semi-quantitative analysis (**F**) (n = 5 animals per group). *p < 0.05 or **p < 0.01 versus sham; ^**#**^*p* < 0.05 or ^**##**^*p* < 0.01 versus vehicle. Cropped blots were used here and the full-length gels were included in the supplementary information.

Oxidative stress is a prominent mediator of MI/R injury by provoking multifactorial damages including mitochondrial dysfunction and apoptosis^23^. We observed that MI/R induced oxidative stress, as evidenced by drastically increased steady-state levels of reactive oxygen species (ROS) (Fig. 5A) and nitrotyrosine content (Fig. 5B,C), while YXS treatment dramatically reduced their levels.

**Figure 6:**
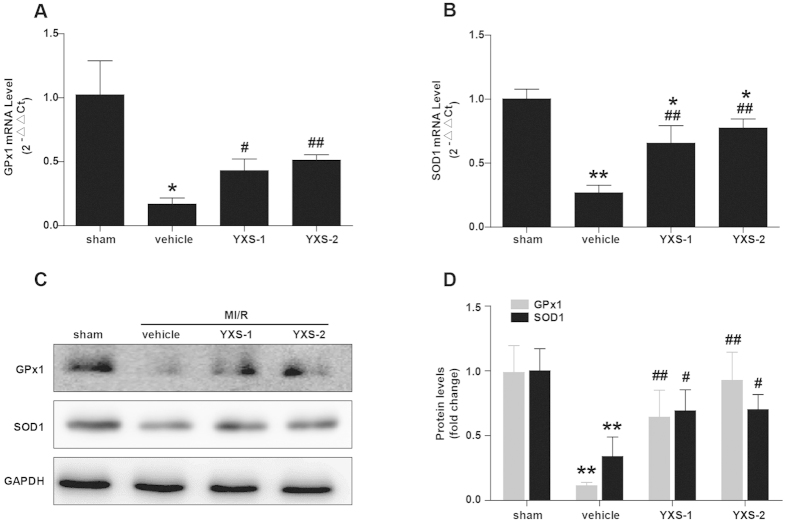
YXS pretreatment normalizes expression of antioxidant enzymes. (**A,B**) The mRNA levels of GPx1 (**A**) and SOD1 (**B**) were determined by real-time quantitative PCR (n = 4 animals per group). (**C,D**) Representative Western blot bands of GPx1, SOD1 (**C**) and semi-quantitative analysis (**D**) (n = 4 animals per group). *p < 0.05 or **p < 0.01 versus sham; ^**#**^*p* < 0.05 or ^**##**^*p* < 0.01 versus vehicle. Cropped blots were used here and the full-length gels were included in the supplementary information.

MI/R-elicited oxidative stress results from augmented reactive species generation and impairment of the antioxidant defense machinery^24^,^25^. We further determined the effects of YXS treatment on pro-/anti-oxidant enzymes. As shown in Fig. 5D–F, YXS significantly inhibited NADPH oxidase activity, and decreased MI/R-induced upregulation of the pro-oxidant enzymes iNOS and gp91^phox^ subunit of the NADPH oxidase. On the other hand, expression levels of the important anti-oxidant enzymes GPx1 and SOD1 were significantly downregulated after MI/R (Fig. 6A–D), while YXS treatment preserved their mRNA and protein levels as compared with vehicle (Fig. 6A–D). Overall, these data indicate that the cytoprotective effect of YXS in MI/R injury is related to oxidative stress abrogation.

### The beneficial effects of YXS are associated with upregulation of LXRα

**Figure 7:**
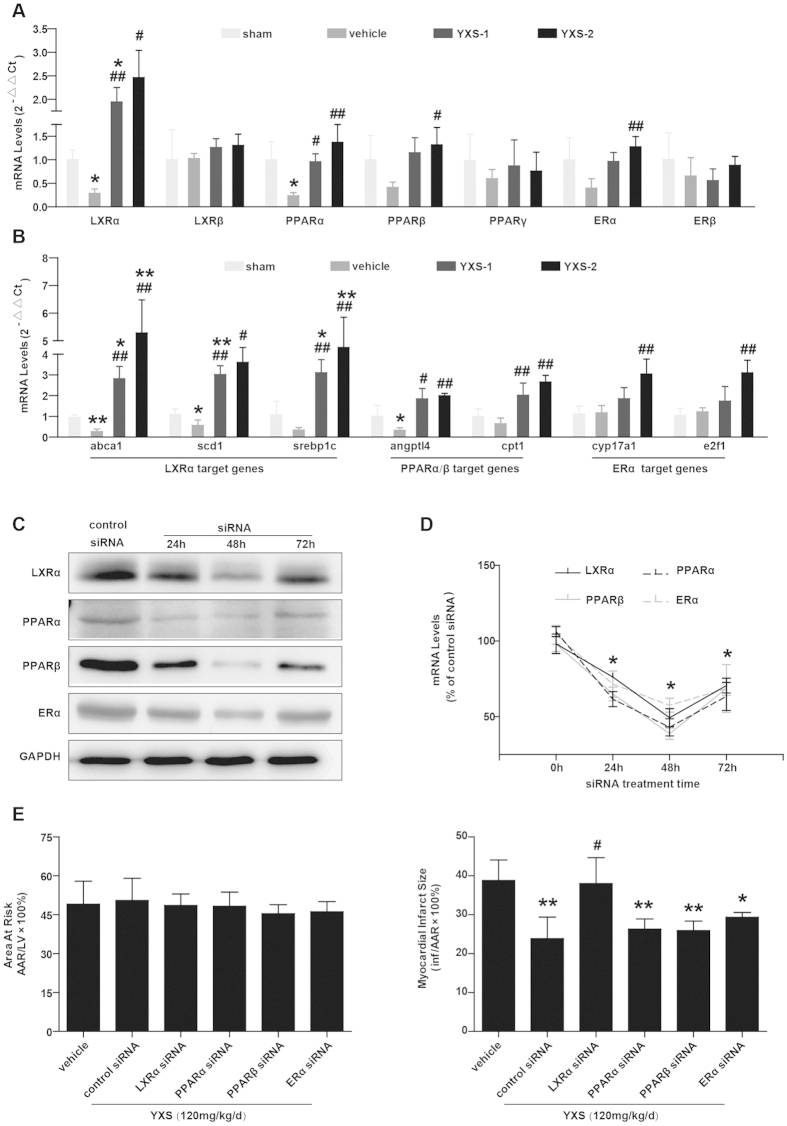
Silencing of cardiac-specific LXRα blunts the beneficial effect of YXS on infarct size. (**A**) YXS pretreatment selectively upregulated expression of LXRα, PPARα, PPARβ and ERα in ischemic/reperfused myocardium compared with vehicle treatment (n = 4 animals per group). *p < 0.05 versus sham; ^**#**^*P* < 0.05 or ^**##**^*p* < 0.01 versus vehicle. (**B**) YXS upregulated expression of target genes of LXRα, PPARα, PPARβ and ERα compared with vehicle treatment (n = 4 animals per group). *p < 0.05 or **p < 0.01 versus sham; ^**#**^*p* < 0.05 or ^**##**^*p* < 0.01 versus vehicle. **C**: Representative Western blot bands showing LXRα, PPARα, PPARβ and ERα protein expression at 24 h, 48 h, and 72 h after intramyocardial siRNA delivery (n = 4 animals per group). (**D**) Real-time q-PCR results showing relative mRNA levels of LXRα, PPARα, PPARβ and ERα after *in-vivo* siRNA-mediated silencing for the indicated time points; results were expressed as fold change over control siRNA delivery (n = 3 animals per group). *p < 0.01 versus control siRNA. (**E**) Quantification of AAR (left panel) and infarct size (right panel) in indicated groups (n = 7 animals per group). *p < 0.05 or **p < 0.01 versus vehicle; ^#^*p* < 0.05 versus control siRNA + YXS. Cropped blots were used here and the full-length gels were included in the supplementary information.

**Figure 8:**
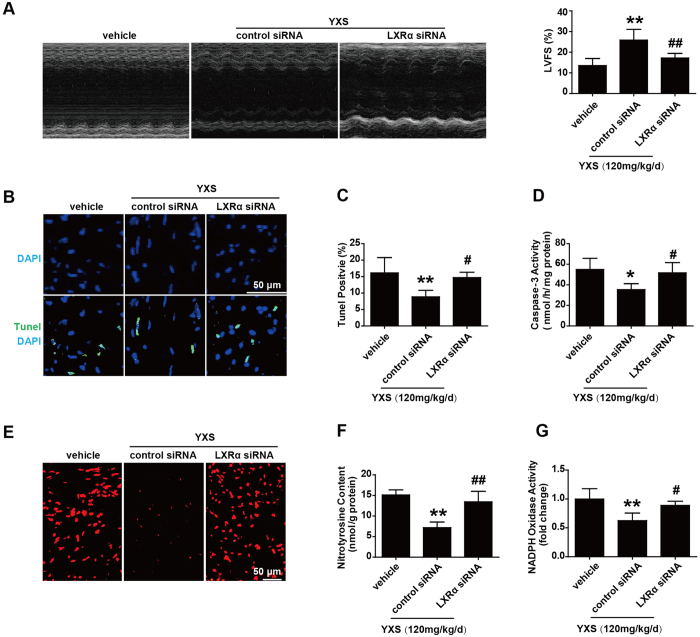
Silencing of cardiac-specific LXRα blunts the beneficial effect of YXS on cardiac dysfunction, apoptosis and oxidative stress. (**A**) Representative images of echocardiography among different groups (left panel). LVFS values (right panel) were subjected to statistical analysis (n = 6 animals per group). (**B**) Representative images of TUNEL staining showing that LXRα depletion blunted the effect of YXS on myocardial apoptosis. (**C**) Quantitative analysis of apoptotic cardiomyocytes among the indicated groups (n = 5 animals per group). (**D**) Caspase-3 activities in indicated groups (n = 5 animals per group). (**E**) ROS steady-state levels in ischemic myocardium were measured by *in-situ* dihydroethidium staining, and images were obtained with confocal microscopy. (**F**) Nitrotyrosine production in ischemic/reperfused myocardium was measured by ELISA (n = 5 animals per group). (**G**) NADPH oxidase activity was measured by lucigenin-enhanced chemiluminescence, and expressed as fold change relative to sham-operated controls (n = 5 animals per group). *p < 0.05 or **p < 0.01 versus vehicle; ^#^*p* < 0.05 or ^##^*p* < 0.01 versus control siRNA + YXS.

Nuclear receptors (NRs), ligand-activated transcription factors that vitally regulate cardiovascular function, are potential targets of certain TCM compounds^12^ (Supplementary Table S1). We thus screened NRs that might be regulated by YXS and mediate its cardio-protective effects. Interestingly, YXS treatment selectively upregulated expression of LXRα, PPARα, PPARβ and estrogen receptor α (ERα) as compared with vehicle treatment (Fig. 7A). Consistently, YXS treatment also led to dramatic upregulation of expression of target genes of LXRα (abca1, scd1, srebp1c), PPARα and PPAR β (angptl4, cpt1), and ERα (cyp17a1, e2f1), respectively (Fig. 7B). Considering the established cardio-protective roles of these NRs^7^,^26^,^27^, we further tested if their upregulation contributed to the effects of YXS. To this end, we performed intramyocardial injection of siRNA to silence the expression of LXRα, PPARα, PPARβ and ERα, respectively, in YXS-treated mice. MI/R was induced when cardiac expression of these NRs reached a nadir, namely 48 hours after siRNA delivery (Fig. 7C,D). Interestingly, only LXRα depletion significantly blunted the protective effects of YXS on MI/R-induced infarct size, while knockdown of PPARα, PPARβ and ERα only slightly blunted the effect of YXS on infarct size (Fig. 7E), suggesting that the favorable effect of YXS was mainly dependent on LXRα upregulation. To further confirm the contributory role of LXRα to YXS-mediated protection, we investigated whether LXRα silencing reversed the protective effect of YXS on MI/R-induced cardiac dysfunction. As shown in Fig. 8A, LXRα silencing significantly diminished the beneficial effects of YXS on cardiac dysfunction. Moreover, LXRα knockdown dramatically reversed the effect of YXS on apoptosis (Fig. 8B–D) and oxidative stress (Fig. 8E–G) after MI/R. Collectively, these data suggest that YXS upregulates endogenous nuclear receptors including LXRα, PPARα, PPARβ and ERα in ischemic/reperfused hearts, with its protective effect being largely dependent on LXRα.

## Discussion

Although reperfusion strategies have significantly reduced ischemia-induced injury and mortality, effective intervention strategies for reperfusion-induced injury remain limited^28^. In the present study, pretreatment with YXS, a SMS-derived TCM formula, significantly reduced infarct size and preserved cardiac function in hypercholesterolemic mice subjected to MI/R injury. These beneficial effects of YXS were associated with suppression of mitochondrial mediated apoptosis and reduction of oxidative stress. Moreover, YXS upregulated expression of endogenous NRs including LXRα, PPARα, PPARβ and ERα compared with vehicle treatment, while the protective effects of YXS were significantly blunted by knockdown of cardiac-specific LXRα. Collectively, these data suggest that YXS is a potent TCM drug which reduces MI/R injury by suppressing mitochondrial mediated apoptosis and oxidative stress and by upregulating LXRα.

Because of the limited regenerative capacity of the myocardium, cardiomyocyte dropout due to apoptosis is a major determinant of MI/R-induced cardiac dysfunction^29^,^30^. Activation of apoptosis pathways (i.e., the death receptor, mitochondrial and ER-stress death pathways) is a prerequisite for MI/R-induced apoptosis^7^. In the present study, YXS attenuated myocardial apoptosis and suppressed the mitochondrial mediated apoptosis pathway, as evidenced by the attenuation of mitochondrial swelling, reduction of mitochondrial cytochrome *c* release and inhibition of caspase-9 activity. In contrast, YXS did not alter the death receptor and endoplasmic reticulum-stress death pathways, suggesting that YXS maintains cardiac survival mainly by suppressing the mitochondrial-mediated apoptosis pathway.

Reperfusion-induced oxidative stress is a key contributor to MI/R injury by inducing a cascade of pathological changes that include mitochondrial dysfunction, DNA damage, and protein aggregation which in turn lead to apoptosis^23^. MI/R-induced redox aberrance results not only from excessive ROS generation but also from antioxidant system impairment^24^,^25^. YXS dampened oxidative stress by counterbalancing pro- and anti-oxidative machineries in the reperfused myocardium. On the one hand, YXS treatment downregulated expression of the pro-oxidative enzymes iNOS and gp91^phox^ subunit of NADPH oxidase, while on the other hand, YXS significantly increased expression of two important antioxidant enzymes (GPx1 and SOD1). Because oxidative stress is a common culprit among many cardiac disorders other than MI/R injury^31^,^32^, findings in the present study provide clues for the potential effects of YXS in other oxidative damage-associated cardiovascular diseases. Of note, in addition to oxidative stress, excessive inflammatory response is similarly important as a mediator by directly promoting myocardial apoptotic response after MI/R^33^. Interestingly, we further observed that YXS significantly reduced neutrophil infiltration in the ischemic area, and reduced serum levels of chemoattractants involved in regulating neutrophil chemotaxis such as CXCL1, CXCL2, CCL5, MIP-1α, and MCP-1 after MI/R (Supplementary Fig. S1), suggesting an anti-inflammatory effect of YXS in the setting of MI/R, which might also contribute to the anti-apoptotic effect of YXS.

In the past decade, the scientific community has focused on clarifying the molecular mode of action of TCM^34^,^35^,^36^,^37^,^38^,^39^. Of note, research on the role of TCM-NRs gained momentum, and many NRs were identified as intracellular targets of TCM ingredients^12^,^38^,^40^. NRs are a family of transcription factors involved in the regulation of diverse biological functions^41^, including cardiac homeostasis maintenance^7^,^42^,^43^. In particular, many NRs such as LXRα and ERs and are pivotal modulators of redox balance and myocyte survival in reperfused hearts^7^,^26^,^27^. Interestingly, previous studies on non-myocyte (e.g., HepG2 and HeLa) cells reported that almost all the individual components of YXS have agonistic activities on one or more of the following NRs: LXRs, PPARs, ERs, progesterone receptor (PR) and pregnane X receptor (PXR) (Supplementary Table S1)^12^,^44^,^45^,^46^,^47^,^48^. However, in the present study, only LXRα, PPARα, PPARβ and ERα, but not PR or PXR, were upregulated by YXS in ischemic/reperfused myocardium, which might be explained by the tissue-specific distribution of NRs in mammals: PR is barely detectable in the heart^49^ and PXR is much less abundantly expressed in the myocardium than in other tissues^50^. Of note, the present study further revealed that siRNA depletion of LXRα significantly diminished the protective effect of YXS, while knockdown of PPARα, PPARβ and ERα only slightly blunted the effect of YXS, suggesting that the action of YXS is mainly dependent on LXRα. In line with this finding, expression of cardiac LXRα and its target genes was upregulated the most by YXS, which is consistent with earlier reports that both “sovereign” ingredients in YXS (*Radix Ginseng* and *Salvia Miltiorrhiza)* are potent agonists of LXRs^12^,^44^. Collectively, our findings provide the first evidence for the association of TCM and NRs in acute ischemic heart injury, and suggest that NRs might be important mediators of the cardio-protective effects of various TCM formulas. Previously, we and others have reported that synthetic LXR agonists are highly effective in attenuating MI/R injury by suppressing oxidative stress^7^. However, severe side effects of LXR agonists such as hepatic steatosis limited their clinical application^51^. YXS treatment might represent a promising alternative means to obtain the benefits of LXRα activation.

Several limitations of the current study deserve to be mentioned. Firstly, although our results demonstrated a beneficial effect of YXS against acute MI/R injury, the long-term beneficial effects of YXS on cardiac remodeling and dysfunction post MI remain unclear. Future studies are warranted to clarify this issue. Secondly, because myocardium contains different cell types, the effect of YXS on individual cell types warrants investigation.

In conclusion, SMS-based YXS appears effective in mitigating acute MI/R injury via suppression of oxidative stress and myocardial apoptosis, in association with upregulation of the endogenous cardio-protective nuclear receptor LXRα. Our data provide important experimental data for further pharmacological research, and a rationale for future clinical trials and applications.

## Methods

### Drug and reagents

YiXin-Shu, extracted and concentrated from a group of herbal medicines including *Panax Ginseng*, *Schisandra Chinensis*, *Ophiopogon Japonicus, Astragalus Membranaceus*, *Salvia Miltiorrhiza*, *Ligusticum Wallichii*, and *Fructus Crataegi* (Supplementary Table S1), was obtained from Xinbang Pharmaceutical Co. Ltd. (Guizhou, China); 4′,6-diamidino-2-phenylindole (DAPI) and 2,3,5-triphenyltetrazolium chloride (TTC) were purchased from Sigma-Aldrich (St. Louis, MO), and dihydroethidium (DHE) and TRIzol Reagent were from Life Technologies (Carlsbad, CA); and in-vivo-jet Polyethylenimine (PEI) was obtained from PolyPlus-Transfection (Illkirch, France).

### Animals and dietary protocols

Six-week old male C57BL/6 mice (21–25 g) were obtained from Slac Laboratory Animal Co., Ltd. The mice were housed in cages at 24 ± 2 °C, humidity of 40 ± 5%, under a 12-hour light/dark cycle, and received standard diet and water ad libitum. Mice were adapted for 2 weeks, then fed with a high-cholesterol diet (prepared by and purchased from Biomodel Organism Science & Technology Development Co., Ltd., Shanghai, China) for 8 weeks, and subsequently randomly assigned to the following groups: sham, vehicle (saline), YXS-1 (60 mg·kg^−1^·d^−1^, equivalent to clinical dosage), or YXS-2 (120 mg·kg^−1^·d^−1^) by gavage for 1 week. At the end of vehicle or YXS treatment, tail vein blood was collected and centrifuged at 4 °C to determine plasma triglyceride (TG), total cholesterol (TC), and low-density lipoprotein-cholesterol (LDL-C) levels using an auto-biochemical analysis system (Chemix-180, Sysmex, Japan). The protocols for *in vivo* study with mice were approved by the Animal Ethics Committee of Shanghai Jiao Tong University, and the methods for *in vivo* study were carried out in accordance with the approved guidelines.

### Surgical procedure

After administration of the last dose of YXS or vehicle, the MI/R model was generated as previously described^42^,^52^. Briefly, mice were anesthetized with 2% isoflurane, with anesthesia considered adequate when the pedal withdrawal reflex was negative. The heart was manually exposed without intubation via a tiny thoracic incision. For myocardial ischemia, a slipknot was tied around the left anterior descending coronary artery (LAD) 3–4 mm from its origin utilizing a 6-0 silk suture. Successful myocardial ischemia was induced when the anterior wall of the LV turned pale. Sham-operated animals were subjected to the same surgical procedures, but the suture passing beneath the LAD was not tied. After 30 minutes of ischemia, the slipknot was released and reperfusion commenced. Upon recovery from surgical procedure, mice were returned to standard animal housing conditions. The surgery was performed by a technician blinded to treatment assignment. No difference was observed in surgical mortality among groups investigated.

### Determination of myocardial infarct size

Myocardial infarct size was determined by Evans blue-triphenyltetrazolium chloride (TTC) double staining^53^ at 24 hours after MI/R. Mice underwent thoracotomy under anesthesia. After heart was arrested at the diastolic phase via KCl injection, the LAD coronary artery was retied with the same suture used for ligation. To demarcate the area at risk (AAR), the ascending aorta was cannulated and perfused with 0.01 ml 2% Evans blue dye. The heart was quickly excised, frozen at −80 °C, and sliced into 1 mm thick cross sections. The heart sections were then incubated individually using a 24-well culture plate with 1% TTC solution at 37 °C for 15 min, and photographed digitally. Infarct size, AAR, and total LV area were measured in each section using Image Pro Plus 6.0. (Bethesda, MD, USA). AAR was expressed as the percentage of AAR over total LV area; and the myocardial infarct size as the ratio of infarct area over AAR.

### Fluorodeoxyglucose micro-positron emission tomography/computed tomography (^18^F-FDG micro-PET/CT scanning)

Viable myocardium metabolism was detected by micro-PET/CT scanning as previously described^7^,^52^. At 24 hours after MI/R, the animals were injected with^18^ F-FDG (0.1 ml with 10 MBq activity via tail vein), maintained in an anesthesia cage filled with 2% isoflurane for 2 hours, and then placed prone on the PET scanner bed near the central field of view and maintained under continuous anesthesia with isoflurane throughout the study. Ten-minute static PET scanning was performed with the Inveon Acquisition Workplace 601 (IAW). The images were reconstructed using a 3-Dimensional Ordered Subsets Expectation Maximum (OSEM3D) algorithm, followed by Maximization/Maximum a Posteriori (MAP) or FastMAP. The 3D regions of interest (ROIs) were drawn over the heart with reference to the CT images, and the tracer uptake was determined using the Inveon Research Workplace software 3.0. The mean standardized uptake value (SUV) of ^18^F-FDG uptake was calculated in each mouse by dividing the relevant ROI activity by the ratio of the injected dose to body weight.

### Echocardiographic measurements

Cardiac function was assessed using an echocardiographic imaging system (Vevo 770, VisualSonic, Toronto, Canada) at 24 hours after MI/R. The mice were anesthetized using 2% isoflurane in O_2_ gas, end-systolic and end-diastolic LV dimensions on the short-axis view were traced at the papillary muscle tips below the mitral valve, and the left ventricular fractional shortening (LVFS) was calculated as previously described^54^.

### *In-situ* detection of apoptosis in reperfused myocardium

At 3 hours after MI/R, ischemic/reperfused myocardium was harvested and fixed in 4% formalin. Terminal deoxynucleotidyl transferase dUTP nick-end labeling (TUNEL) was carried out to assess myocardial apoptosis using the Fluorescein *In Situ* Cell Death Detection Kit (Roche Diagnostics, Indianapolis, IN) as described previously^55^. Cardiomyocytes, apoptotic nuclei and total cardiomyocyte nuclei were labeled with anti-α-actin antibody, green fluorescein staining and DAPI, respectively. For each slice, 5 fields were randomly obtained with a Leica laser scanning confocal microscope (Leica TCS SP5 II). Extent of cell apoptosis was expressed as ratio of TUNEL positive nuclei over DAPI-stained nuclei.

### Transmission electron microscopy

At 3 hours after MI/R, ischemic myocardium was dissected and fixed with 2% glutaraldehyde for 2 h, then fixed with 1% osmium tetroxide, and embedded as monolayers in LX-112 (Ladd Research). Sections were stained in uranyl acetate and lead citrate. Cardiomyocyte mitochondria were observed with an electron microscope (Philips CM-120; Philips Electronic Instruments).

### Determination of caspase activities

Caspase-3 activity was measured with caspase-3 Colorimetric Assay Kit (Millipore, Billerica, MA) as previously described^56^. Briefly, at 3 hours after MI/R, hearts were excised and homogenized, and 100 μg of total protein from heart tissues was loaded onto each well of a 96-well plate, and incubated with 25 μg Ac-DEVD-pNA as a colorimetric-specific substrate at 37 °C for 90 min. Caspase-3 cleaved pNA from DEVD, and the free pNA was quantified with a microplate reader (BioTek, Winooski, VT) at 405 nm. Caspase-3 activity was expressed as nanomoles of pNA formation per hour per milligram of protein. Activation of caspase-8, -9, and -12 was tested via corresponding caspase Fluorometric Assay Kits (BioVision, Mountain View, CA) according to manufacturer’s instructions. In brief, 100 μg of total protein from tissues was loaded and incubated with AFC-conjugated substrates (at a final concentration of 50 μM) specific for caspase-8, -9, and -12 (IEDT, LEHD, and ATAD, respectively). Samples were read by a fluorimeter (excitation wavelength, 400 nm; emission wavelength, 508 nm). Caspase-8, -9, and -12 activities were expressed as fold change against sham group.

### Measurement of superoxide generation in reperfused myocardium

Dihydroethidium (DHE) staining was used for *in situ* superoxide detection at 3 hours after MI/R^57^,^58^. Briefly, unfixed frozen slices (5 μm) of ischemic/reperfused myocardium were incubated with DHE (5 μmol/L) at 37 °C for 30 min in a humidified chamber protected from light, and washed with PBS for 5 min. Ethidium staining (red) was confirmed and obtained with a Leica laser scanning confocal microscope. NADPH oxidase activity was measured by lucigenin-enhanced chemiluminescence as previously described^52^. Superoxide production was calculated as relative light units (RLU) per mg per second, and expressed as fold change against sham group.

### Detection of nitrotyrosine levels in reperfused myocardium

At 3 hours after MI/R, nitrotyrosine content was determined quantitatively and qualitatively by enzyme-linked immunosorbent assay (ELISA) analysis and immunohistochemistry, respectively. ELISA was performed using a commercially available kit (Abnova, Taiwan). Briefly, samples were incubated in antibody-coated plates for 1 h at room temperature, followed by sequential incubations with biotinylated tracer IgG and streptavidin horseradish peroxidase complex. After washing with PBS, color development was initiated with tetramethylbenzidine (TMB) substrate for 30 min at room temperature. Reaction was terminated by citric acid. The concentration of nitrotyrosine of each sample was determined against the standard curve. The results are presented as nanomole of nitrotyrosine per gram of protein. For immunostaining, paraffin-embedded slices were stained with primary antibody against nitrotyrosine (1:100, Millipore, Billerica, MA). The immunostaining was performed with the Vectastain ABC kit (1:200, Vector Laboratories, Burlingame, CA), and the slides were photographed in light microscopy.

### *In-vivo* cardiac-specific gene silencing

*In-vivo* cardiac-specific knockdown of LXRα, PPARα, PPARβ and ERα was achieved by intramyocardial injection of siRNA as described in our previous study^7^. Briefly, respective siRNAs targeting LXRα, PPARα, PPARβ, or ERα (GenePharma, Shanghai, China), and control nonspecific siRNA were complexed with *in-vivo* jet-PEI delivery reagent in 5% glucose. After confirmation of adequate anesthesia by the absence of the pedal withdrawal reflex, the mice heart was exposed via an incision made between the fourth and fifth left ribs, and corresponding siRNAs (20 μl; 1 μg/g) were delivered via three separate intramyocardial injections into the left ventricle apex and anterolateral wall.

### Western blot analysis

Cardiac tissue was homogenized in lysis buffer and the supernatant fluid was collected. Mitochondria Isolation Kit (Thermo Scientific) was used to separate cytosolic and mitochondrial fractions, and protein lysate concentrations were determined via Pierce BCA Protein Assay Kit (Thermo Scientific, Rockford, IL). Equal quantities of proteins (20–40 μg/lane) were subjected to 10 or 12% SDS-PAGE, depending upon the molecular weight of target proteins, and transferred onto nitrocellulose membranes. After blocking with 5% bovine serum albumin (BSA), membranes were incubated with primary antibodies against cytochrome c (CST, #4280; 1:1000), Bax (Abcam, ab7977; 1:500), Bcl-2 (Santa Cruz, sc492; 1:500), CHOP (CST, #2895; 1:1000), FAS (Santa Cruz, sc1023; 1:1000), VDAC (CST, #4866; 1:1000), gp91^phox^ (Santa Cruz, sc74514; 1:200), iNOS (Abcam, ab3523; 1:2000), GPx1 (Santa Cruz, sc22145; 1:1000), SOD1 (Santa Cruz, sc101523; 1:1000), LXRα (Abcam, ab41902; 1:1000), PPARα (Santa Cruz, sc9000; 1:2000), PPARβ (Santa Cruz, sc1987; 1:500), and ERα (Santa Cruz, sc787; 1:1000) and GAPDH (CST, #2118; 1:1000). After incubation with the corresponding secondary antibodies, the protein bands were visualized with enhanced chemiluminescence (Millipore, Billerica, MA), and quantitation was performed with Gel-Pro Analyzer 4.0 software (Media Cybernetics, Silver Spring, MD).

### Real-time quantitative PCR

Total RNA was isolated from heart tissues with TRIzol Reagent^59^ and purified with the RNeasy Mini Kit (Qiagen). Complementary DNA (cDNA) was synthesized with Omniscript RT Kit (Qiagen) in accordance with the manufacturer’s instructions. The resultant cDNA was amplified with SYBR^®^ Premix Ex Taq™ Perfect Real-time Kit (Takara BIO, Otsu, Japan) and detected by The LightCycler^®^ 480 Real-Time PCR System (Roche Applied Science, Indianapolis, IN). Ct values, defined as the crossing threshold of PCR, were obtained via LightCycler 480 Data Analysis software. Quantification was performed by the 2^–ΔΔCt^ method^60^. The primer sequences utilized are listed in Supplementary Table S2.

### Statistical analysis

All values are presented as mean ± SD of indicated number of independent experiments. Statistical significance of multiple treatments was determined by one-way analysis of variance (ANOVA) followed by either the Bonferroni *post hoc* test if homogeneity of variance was met or the Tamhane *post hoc* test. All statistical analyses were performed with SPSS software, version 13.0. A 2-tailed P value < 0.05 was considered as statistically significant.

## Additional Information

**How to cite this article**: Zhao, Y. *et al.* YiXin-Shu, a ShengMai-San-based traditional Chinese medicine formula, attenuates myocardial ischemia/reperfusion injury by suppressing mitochondrial mediated apoptosis and upregulating liver-X-receptor α. *Sci. Rep.* **6**, 23025; doi: 10.1038/srep23025 (2016).

## Supplementary Material

Supplementary Information
