# Supplementary material for: YiXin-Shu, a ShengMai-San-based traditional Chinese medicine formula, attenuates myocardial ischemia/reperfusion injury by suppressing mitochondrial mediated apoptosis and upregulating liver-X-receptor α
Source: Sci Rep. 2016 Mar 11;6:23025. doi: 10.1038/srep23025 (PMC4786861; doi:10.1038/srep23025)
Supplement: Supplementary Information [file srep23025-s1.pdf]

YiXin-Shu, a ShengMai-San-based traditional Chinese medicine formula, attenuates myocardial ischemia/reperfusion injury by suppressing mitochondrial mediated apoptosis and upregulating liver-X-receptor α

Yichao Zhao, Longwei Xu, Zhiqing Qiao, Lingchen Gao, Song Ding, Xiaoying Ying, Yuanyuan Su, Nan Lin, Ben He, Jun Pu

Methods and material

Determination of neutrophil recruitment

Hearts were fixed with 4% paraformaldehyde, embedded in paraffin and sectioned into 5-μm slices. After deparaffinization and antigen retrieval, slides were stained with Ly-6G antibody (1:50; Abcam, Cambridge, UK) for neutrophils, and DAPI was used for nuclei visualization. For neutrophil quantification, five fields per heart were taken in the ischemic area and neutrophil number was quantified using digital image analysis software (Image-Pro Plus 6.0, Media Cybernetics, Silver Spring, MD).

Determination of serum levels of chemoattractants

Serum levels of chemoattractants including CXCL1, CXCL2, MIP-1α, MCP-1 and CCL5 injury were measured at 3 hours after MI/R by colorimetric enzyme-linked immunosorbent assay (R&D Systems, Minneapolis, Minnesota).

Supplementary Fig. S1

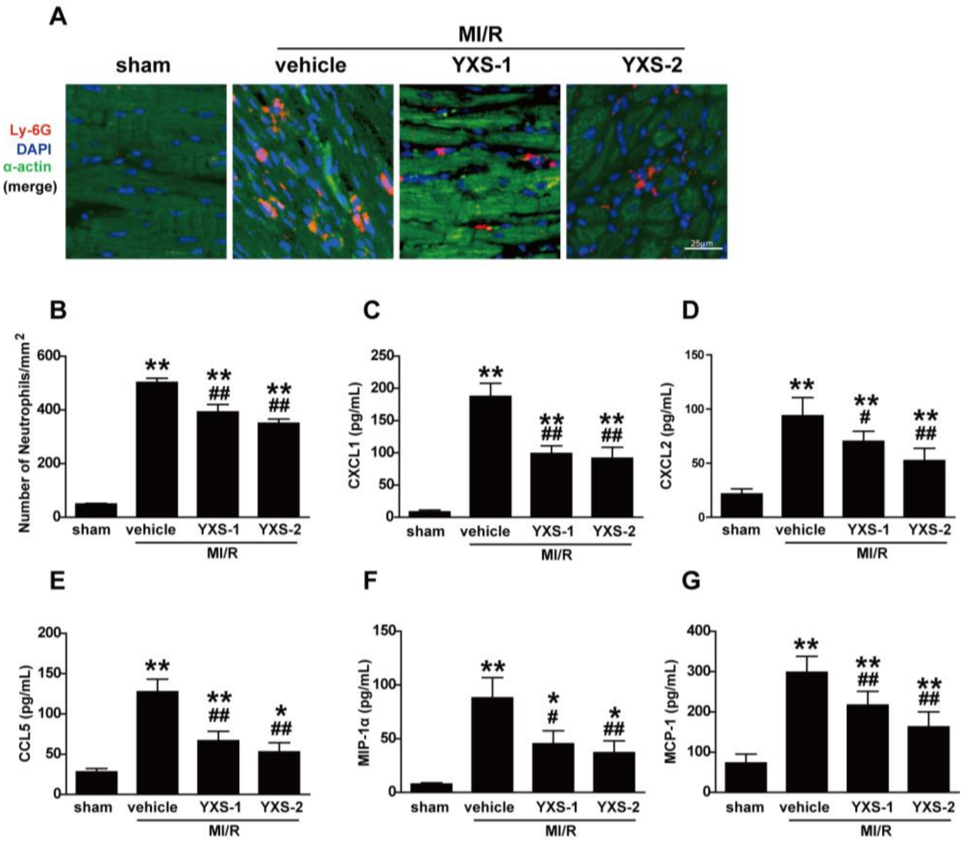

Supplementary Fig. S1. Effects of YXS on MI/R-induced inflammatory response. A. Representative images of immunofluorescence staining for neutrophils in ischemic myocardium. Red, Ly-6G positive neutrophils; blue, total nuclei; green, myocardium. B. Quantification of infiltrated neutrophils in the ischemic myocardium after MI/R among the indicated groups. (n=5). C-G. Serum levels of CXCL1 (C), CXCL2 (D), CCL5 (E), MIP-1α (F), and MCP-1 (G) in the indicated groups (n=5). **P*<0.05 or ***P*<0.01 versus sham group; $^{\#}p<0.05$ or $^{\#\#}p<0.01$ versus vehicle group.

Supplementary Table S1. YXS components and their target nuclear receptors (NRs)

	Latin name	Chinese name	NRs
**Sovereign**	*Radix Ginseng*	Ren Shen	ER (1, 2) LXR (3)
	Salvia Miltiorrhiza	Dan Shen	PPAR (1), LXR (1)
**Minister**	*Astragalus Membranaceus*	Huang Qi	PPAR (4)
	Radix Ophiopogonis	Mai Dong	PXR (5)
	Ligusticum Wallichii	Chuan Xiong	PR (6), PXR (5)
**Assistant**	*Fructus Schisandrae Chinensis*	Wu Wei Zi	PXR (1, 5)
	Fructus Crataegi	Shan Zha	

Supplementary Table S2. Primer sequences used for real-time quantitative PCR

**Gene Name**	**Abbreviation**	**Species**	**Forward (5’-3’)**	**Reverse (5’-3’)**
Glutathione Peroxidase 1	*Gpx1*	mouse	TGCTCATTGAGAATGTCGCGTCTC	AGGCATTCCGCAGGAAGGTAAAGA
Superoxide Dismutase 1	*Sod1*	mouse	ACTAGTATGGCGATGAAAGCGGTG	GGATCCTGTTTACTGGGCAATCCC
Liver X Receptor α	*Lxrα*	mouse	GCTCATTGCCATCAGCAT	AGCATCCGTGGGAACATCA
Liver X Receptor β	*Lxrβ*	mouse	TGCCAGGGTTCTTGCAGTTG	AACGTGATGCATTCTGTCTCGTG
Peroxisome Proliferator-activated Receptor α	*Pparα*	mouse	GTGGTAGCCATTGGCCTTGT	GCCCGGACAGCTTCCTAAGT
Peroxisome Proliferator-activated Receptor β	*Pparβ*	mouse	TCGGGCTTCCACTACGG	ACTGACACTTGTTGCGGTTCT
Peroxisome Proliferator-activated Receptor	*Pparγ*	mouse	ATCGAGGACATCCAAGAC	CAATCTGCCTGAGGTCTG

γ				
Estrogen Receptor α	*Erα*	mouse	GGCCTGACTCTGCAGCAGCAG	GTTGGGGAAGCCCTCTGCTTC
Estrogen Receptor β	*Erβ*	mouse	GGCATTCTACAGTCCTGCTG	TCTGCATAGAGAAGCGATGA
ATP-binding Cassette Transporter A 1	*Abca1*	mouse	CGTTTCCGGGAAGTGTCCTA	GCTAGAGATGACAAGGAGGATGGA
Stearyl CoA Desaturase 1	*Scd1*	mouse	ATGACGTGTACGAATGGGC	GAATGACGTGTACGAATGGGC
Sterol regulatory element-binding protein-1c	*Srebp1c*	mouse	GGAGCCATGGATTGCACATT	GCTTCCAGAGAGGAGGCCAG
Angiopoietin-like 4	*Angptl4*	mouse	GGACTGGGATGGCAATGC	CCTCACCCCCCAAATGG
Carnitine Palmitoyltransferase 1	*Cpt1*	mouse	GATGCAGGAAACTACACGGTCA	GAGATCAAGGCTTTCTCACCGA
Cytochrome P450, Family 17, Subfamily	*Cyp17a1*	mouse	AGTCAAAGACACCTAATGCCAAG	ACGTCTGGGGAGAAACGGT

A, Polypeptiode 1				
E2F Transcription Factor 1	*E2f1*	mouse	ACTCCTCGCAGATC GTCATC	CAGCCTCCGTTTCA CCG
Glyceraldehyde -3-phosphate Dehydrogenase	*Gapdh*	mouse	TGTGTCCGTCGTGG ATCTGA	TTGCTGTTGAAGTC GAAGGAG

Reference

(1) Li, L., Bonneton, F., Chen, X. Y. & Laudet, V. Botanical compounds and their regulation of nuclear receptor action: The case of traditional Chinese medicine. *Mol Cell Endocrinol.* **401**, 221-237 (2015).

(2) Gray, S. L., Lackey, B. R., Tate, P. L., Riley, M. B. & Camper, N. D. Mycotoxins in root extracts of American and Asian ginseng bind estrogen receptors alpha and beta. *Exp Biol Med* (Maywood). **229**, 560-568 (2004).

(3) Kawase, A. *et al.* Increased effects of ginsenosides on the expression of cholesterol 7alpha-hydroxylase but not the bile salt export pump are involved in cholesterol metabolism. *J Nat Med.* **67**, 545-553 (2013).

(4) Shen, P., Liu, M. H., Ng, T. Y., Chan, Y. H. & Yong, E. L. Differential effects of isoflavones, from Astragalus membranaceus and Pueraria thomsonii, on the activation of PPARalpha, PPARgamma, and adipocyte differentiation in vitro. *J Nutr.* **136**, 899-905 (2006).

(5) Yu, C., Chai, X., Yu, L., Chen, S. & Zeng, S. Identification of novel pregnane X receptor activators from traditional Chinese medicines. *J Ethnopharmacol.* **136**, 137-143 (2011).

(6) Lim, L. S., Shen, P., Gong, Y. H. & Yong, E. L. Dimeric progestins from rhizomes of Ligusticum chuanxiong. *Phytochemistry.* **67**, 728-734 (2006).

Full-length gels

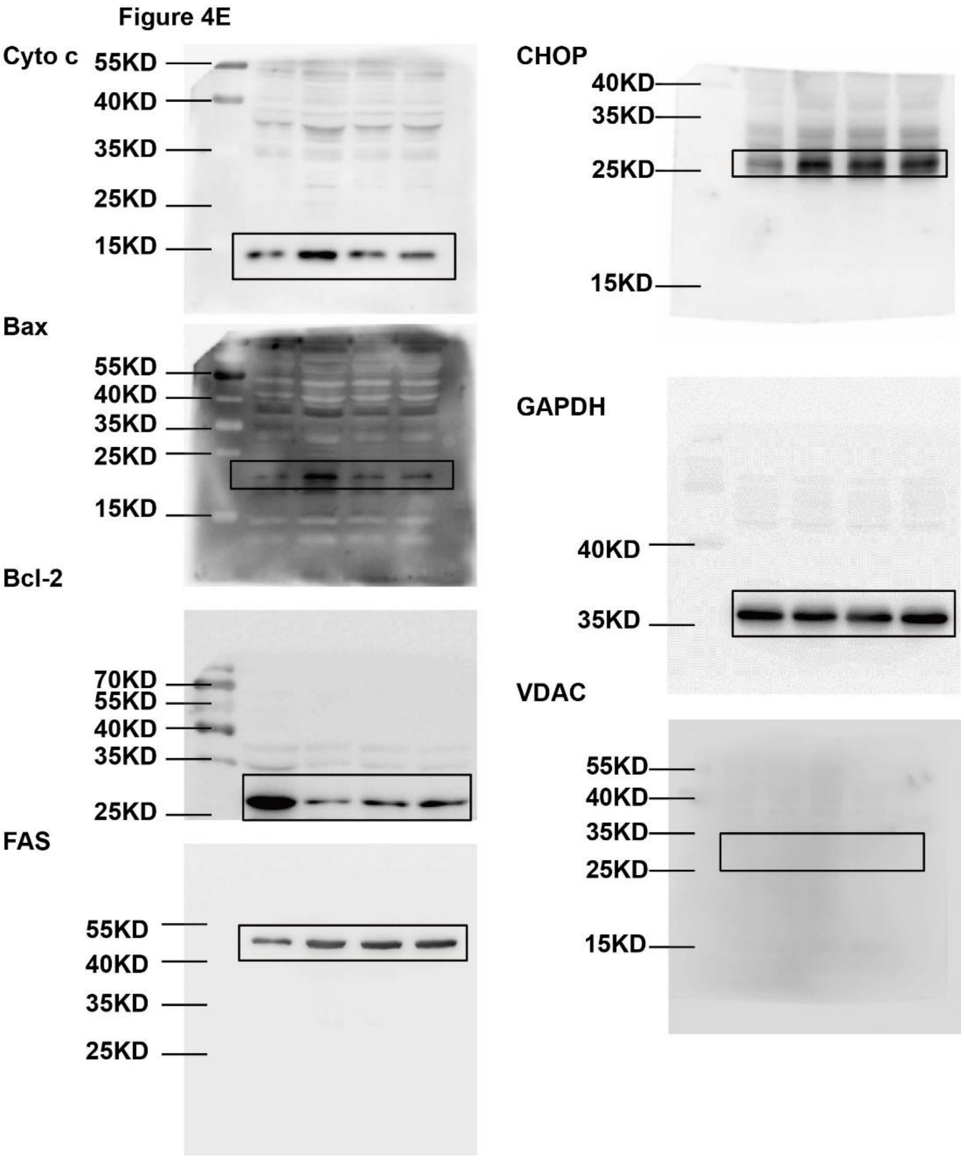

Figure 5E

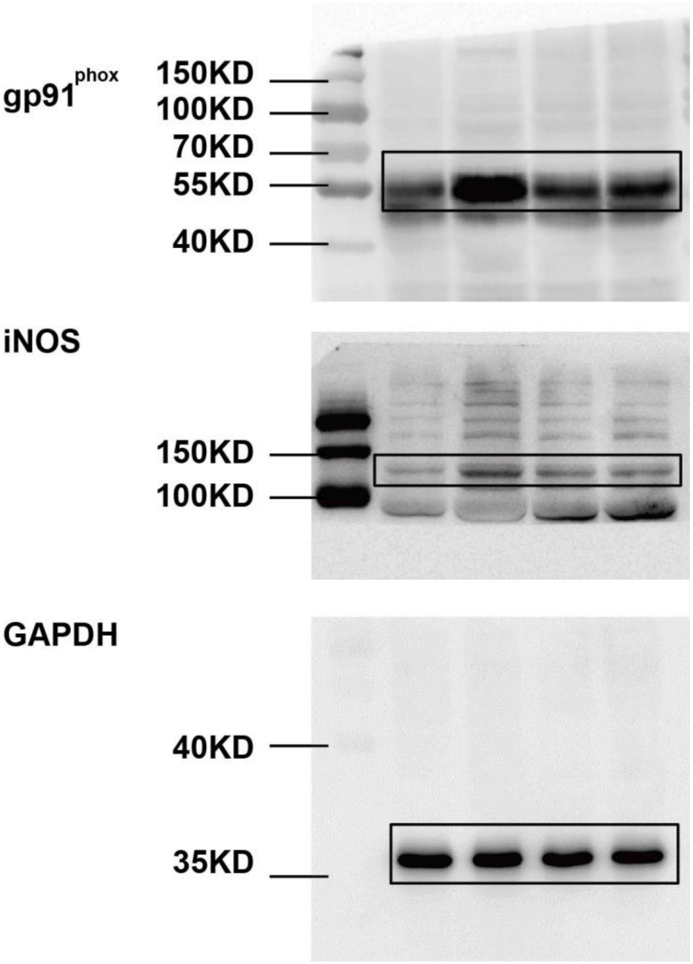
gp91phox
150KD
100KD
70KD
55KD
40KD
iNOS
150KD
100KD
GAPDH
40KD
35KD

Figure 6C

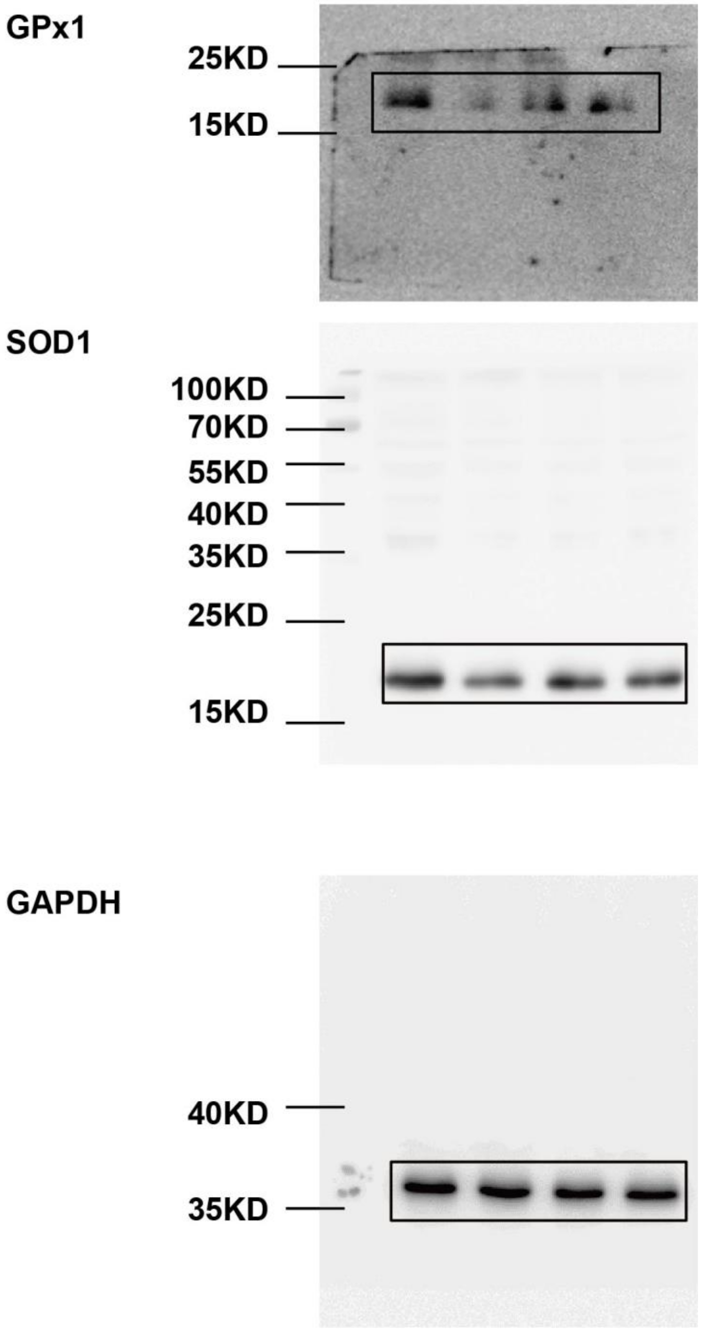

GPx1
25KD
15KD
SOD1
100KD
70KD
55KD
40KD
35KD
25KD
15KD
GAPDH
40KD
35KD

Figure 7C

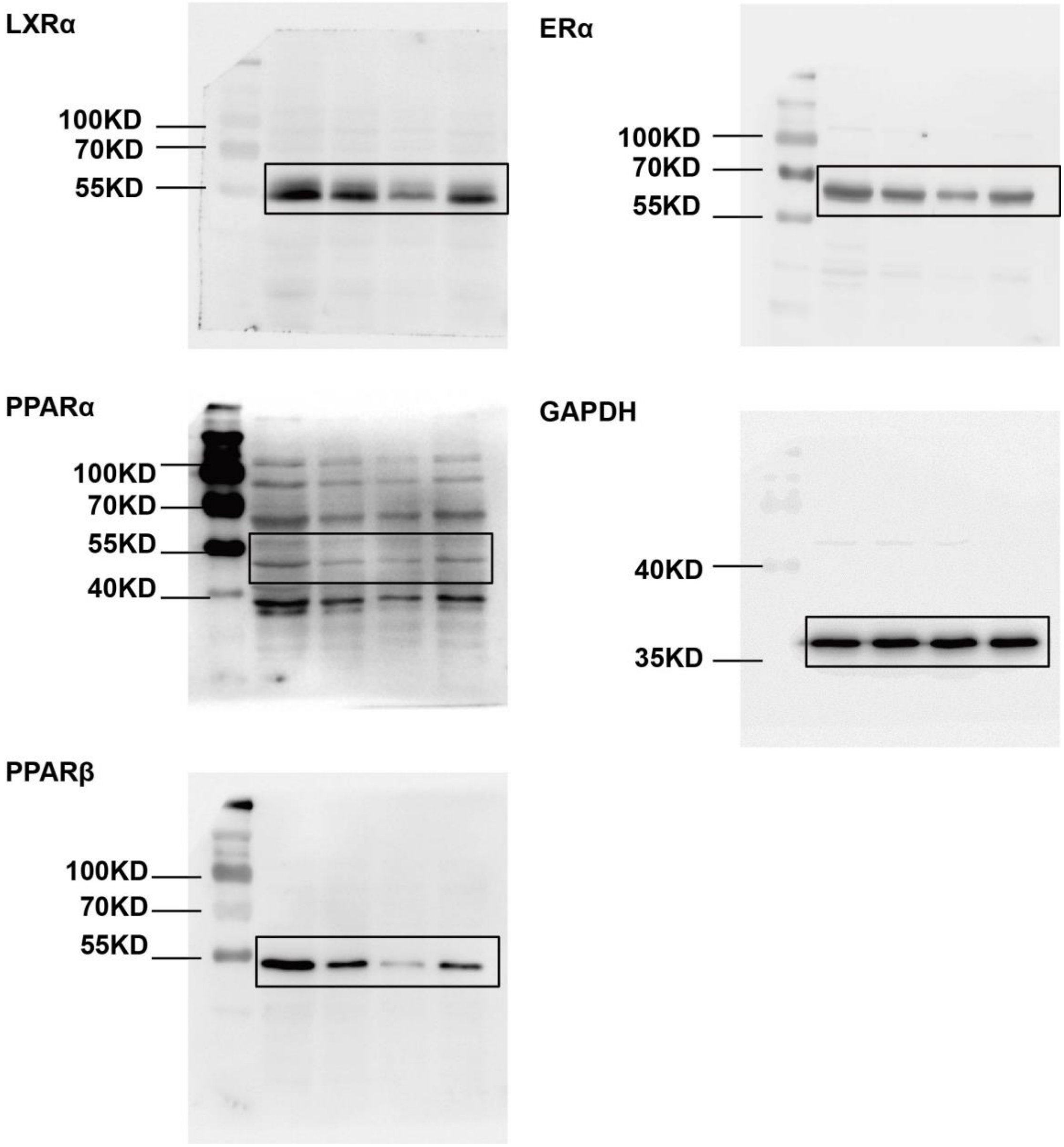

LXRα
100KD
70KD
55KD
ERα
100KD
70KD
55KD
PPARα
100KD
70KD
55KD
40KD
GAPDH
40KD
35KD
PPARβ
100KD
70KD
55KD
